# Association between Metabolically Healthy Obesity and Subclinical Atherosclerosis in the Cardiovascular and Metabolic Diseases Etiology Research Center (CMERC) Cohort

**DOI:** 10.3390/jcm11092440

**Published:** 2022-04-26

**Authors:** Da Hea Seo, Yongin Cho, Seongha Seo, Seong Hee Ahn, Seongbin Hong, Kyung Hwa Ha, Jee-Seon Shim, Hyeon Chang Kim, Dae Jung Kim, So Hun Kim

**Affiliations:** 1 Department of Endocrinology and Metabolism, Inha University College of Medicine, Incheon 22212, Korea; cellory@gmail.com (D.H.S.); choyorin@gmail.com (Y.C.); scolscol@naver.com (S.S.); shahn1017@inha.ac.kr (S.H.A.); sbhongmd@inha.ac.kr (S.H.); 2 Department of Endocrinology and Metabolism, Ajou University School of Medicine, Suwon 16499, Korea; khha84@gmail.com; 3 Department of Preventive Medicine, Yonsei University College of Medicine, Seoul 03722, Korea; shimjs@yuhs.ac (J.-S.S.); hckim@yuhs.ac (H.C.K.)

**Keywords:** metabolic syndrome, obesity, atherosclerosis

## Abstract

We aimed to investigate the association between a new definition of metabolic health (MH) and subclinical atherosclerosis in a cohort of patients without previous cardiovascular disease (CVD). In total, 7824 community-dwelling adults were categorized as normal weight, overweight, or obese. Metabolically healthy obesity (MHO) was defined as obesity accompanied by all of the following criteria: systolic blood pressure (BP) < 130 mmHg, no use of BP-lowering medication, waist-hip ratio <0.832 (women) and <0.887 (men), and no prevalent diabetes. Carotid atherosclerosis was defined as carotid plaque or mean carotid intima-media thickness ≥ 1.1 mm. The prevalence of carotid atherosclerosis was 8.3% and 1113 (14.2%) patients were classified as having MHO. All individuals classified as metabolically unhealthy were at an increased risk of carotid atherosclerosis independent of body mass index categories. Conversely, the risk of carotid atherosclerosis in individuals with MHO was not significantly increased compared to that in metabolically healthy normal weight participants (hazard ratio 1.20, 95% confidence interval 0.87–1.67). This new definition of MH was able to identify people with MHO without an increased risk of CVD in an Asian community cohort.

## 1. Introduction

The prevalence of obesity continues to rise exponentially worldwide and is extensively associated with multiple comorbidities, such as cardiovascular diseases (CVD), diabetes, hypertension, and several cancers [1,2,3]. Obesity is known to accelerate early atherosclerotic changes [4] and is a well-recognized independent risk factor for CVD [5]. Nevertheless, not all people with obesity have an increased risk of cardiometabolic diseases. Recently, there has been growing interest in this unique subgroup of obese (OB) individuals who do not have metabolic abnormalities, referred to as metabolically healthy obesity (MHO), despite their increased adiposity [6].

Studies on MHO and CVD have shown inconsistent results, as the diagnostic criteria varied among the studies [7,8,9,10,11,12]. However, recent meta-analyses and large-scale cohort studies have observed increased risks for CVD mortality and total mortality in individuals with MHO, despite the absence of metabolic syndrome (MetSyn) [9,13,14,15]. In other words, current definitions and criteria for metabolic health (MH) may not be sensitive enough to identify an OB subgroup without an increased risk of CVD. Recently, a new definition of MH has been proposed using anthropometric and metabolic parameters associated with CVD mortality in the third National Health and Nutrition Examination Survey (NHANES-III), which was validated in an independent, population-based prospective cohort [16]. This newly proposed definition of MH was able to identify a subgroup of people with obesity without an increased risk of CVD mortality [16]. However, the majority of the study population was Caucasian, and the Asian population was underrepresented. Therefore, we aimed to evaluate the association between this new definition of MH and subclinical atherosclerosis in an Asian cohort of middle-aged adults without known CVD and compare it with a commonly used definition of MH.

## 2. Materials and Methods

### 2.1. The Study Population

The Cardiovascular and Metabolic Diseases Etiology Research Center (CMERC) cohort consisted of community-dwelling Korean adults aged 30–64 years who were free from myocardial infarction, heart failure, or stroke. All participants completed health questionnaires and examinations. The sampling and measurement procedures in the CMERC cohort have been described in detail previously [17,18]. Briefly, this cross-sectional study collected baseline data of 7824 participants from the CMERC cohort between December 2013 and March 2018. For this analysis, participants with a body mass index (BMI) less than 18 or those who did not undergo carotid artery ultrasonography examination were excluded. This study was approved by the institutional review boards of Severance Hospital, Yonsei University Health System, Seoul, Korea (4-2013-0661) and Ajou University Hospital, Suwon, Korea (AJIRB-BMR-SUR-13-272). All participants provided written informed consent.

### 2.2. Anthropometry Measurements

Every participant’s height and body weight were measured while they wore light clothing and without shoes and were measured to the nearest 0.1 cm or 0.1 kg. BMI was calculated as body weight in kilograms divided by the square of standing height in meters. Waist circumference (WC) was measured at the midpoint between the lower point of the rib cage and the upper point of the iliac crest. Hip circumference was measured in the widest region of the hip.

### 2.3. Laboratory Assays

Blood samples were collected from the antecubital vein after overnight fasting. All analyses were performed at a single laboratory center (Seoul Clinical Laboratories R&D Center, Seoul, Korea). Fasting blood glucose and creatinine levels were determined using a colorimetry method (ADVIA 1800; Siemens, Tarrytown, NY, USA). Hemoglobin A1c (HbA1c) levels were determined using high-performance liquid chromatography with a Variant II Turbo (Bio-Rad Laboratories, Hercules, CA, USA). Fasting insulin levels were determined using a radioimmunoassay with an SR-300 apparatus (Stratec; Birkenfeld, Rhineland-Palatinate, Germany). Total cholesterol, high-density lipoprotein (HDL) cholesterol, low-density lipoprotein (LDL) cholesterol, and high-sensitivity C-reactive protein (hs-CRP) levels were assayed by enzymatic methods (ADVIA 1800; Siemens). To evaluate insulin resistance, the homeostasis model assessment of insulin resistance (HOMA-IR) was calculated using the following formula: [fasting glucose (mg/dL) × fasting insulin (μIU/mL)]/405 [19]. The Chronic Kidney Disease-Epidemiology equation was used to calculate the estimated glomerular filtration rate (eGFR) [20].

### 2.4. Definition of MH and OB Phenotype

Participants were classified as “metabolically healthy” or “metabolically unhealthy” according to the recently proposed definition of MH by Zembic et al., which consists of three cardiometabolic components [16], with modification for waist-hip ratio (WHR) using optimal cutoff values previously defined for Koreans [21]. A person was “metabolically healthy” when all three of the following criteria were fulfilled: (1) systolic blood pressure (SBP) less than 130 mmHg and no use of blood pressure-lowering medication; (2) WHR <0.832 (women) or <0.887 (men); and (3) no prevalent diabetes. We also compared this new definition with the most widely used criteria for MetSyn, the National Cholesterol Education Program–Adult Treatment Panel III (NCEP-ATPIII) criteria (NCEP definition). Individuals were considered metabolically healthy if they were free of MetSyn components: WC greater than 90 cm (men) or greater than 85 cm (women); BP ≥ 130/85 mmHg or using blood pressure-lowering medication; triglyceride (TG) level ≥ 150 mg/dL or use of lipid-lowering medication; high-density lipoprotein cholesterol level < 40 mg/dL (men) or <50 mg/dL (women); and fasting glucose level ≥100 mg/dL or prevalent diabetes [22,23,24].

BMI was classified as normal weight (NW) (<23 kg/m^2^), overweight (OW) (23 to <25 kg/m^2^), OB class 1 (25 to <30 kg/m^2^) or OB class 2 (≥30 kg/m^2^) [25]. We evaluated six mutually exclusive body size phenotypes based on metabolic status and BMI categories: metabolically healthy normal weight (MHNW), metabolically unhealthy normal weight (MUHNW), metabolically healthy overweight (MHOW), metabolically unhealthy overweight (MUHOW), metabolically healthy obese (MHO), and metabolically unhealthy obese (MUHO).

### 2.5. Assessment of Carotid Atherosclerosis

Carotid artery ultrasonography was performed using high-resolution ultrasonographic systems (Accuvix XG, Samsung Medison, Seoul, Korea and Logiq S8, GE Healthcare, Chalfont St. Giles, UK). All operators were trained according to a predefined protocol. Carotid intima-media thickness (CIMT) was scanned at the 1-cm segment of the common carotid arteries proximal to the bulb region. The mean and maximum CIMT values were measured on both the right and left sides. A carotid artery plaque was considered present if the thickness was greater by 50% or more than the surrounding intima-media thickness (IMT), or if its size was ≥1.5 mm [26]. Carotid atherosclerosis was defined as carotid plaque or mean CIMT ≥ 1.1 mm [27].

### 2.6. Assessment of Nonalcoholic Fatty Liver Disease (NAFLD)

The hepatic steatosis index (HSI) was used to define NAFLD, and those with HSI > 36 were considered to have NAFLD: HSI = 8 × (ALT/AST ratio) + BMI (+2, if female; +2, if diabetes mellitus) [28].

### 2.7. Statistical Analyses

Data are presented as mean ± standard deviation or median (interquartile range) for continuous variables and as numbers (percentages) for categorical variables. Comparisons among the three groups according to obesity status (normal weight, overweight and obesity) were performed by using one-way analysis of variance with Bonferroni-corrected post hoc comparisons (for normal distribution) or Kruskal-Wallis tests with post hoc pairwise comparisons (for nonnormal distribution). Due to the right-skewed distribution, HOMA-IR, TG, and hs-CRP levels were entered into the models as log-transformed units. Multivariate logistic regression analyses were performed to examine the independent association of obesity phenotypes with carotid atherosclerosis or NAFLD after adjustment for age, sex, TG, HDL-C, LDL-C, hsCRP, HbA1c, HOMA-IR, eGFR, smoking status, alcohol consumption, physical activity, and the other definition of MH according to the NCEP-ATP III criteria. We repeated the same analysis using MH definition by NCEP-ATP III. All statistical analyses were performed using SPSS software (version 26.0; IBM Corp., Armonk, NY, USA). Statistical significance was defined as a two-sided *p*-value less than 0.05.

## 3. Results

**Figure 1:**
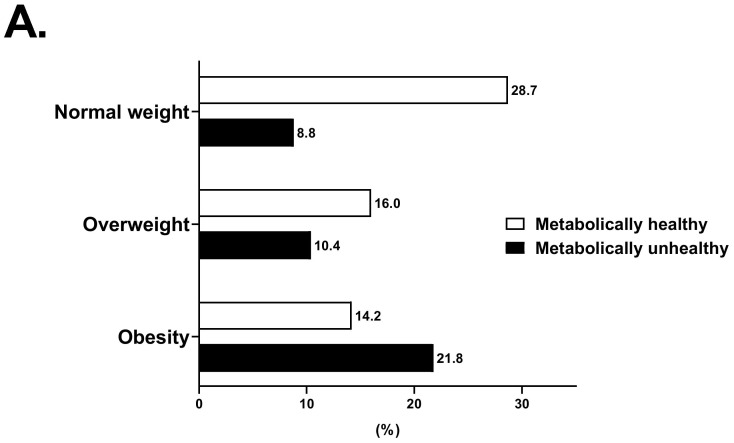

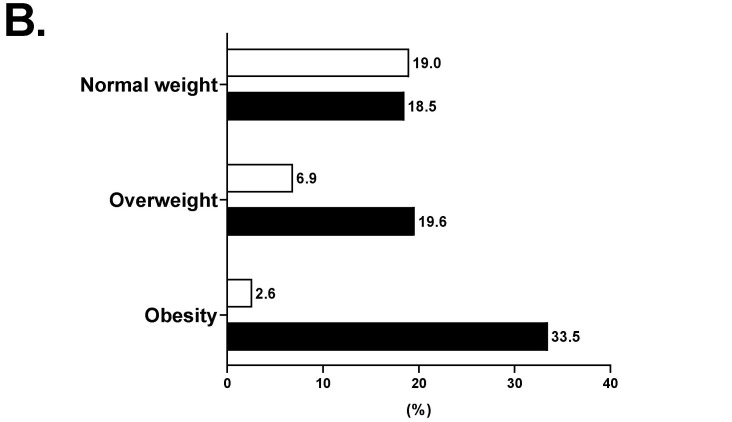
Prevalence of different body phenotypes across categories of weight and metabolic status: based on new definition (**A**) and based on the National Cholesterol Education Program–Adult Treatment Panel III (NCEP-ATPIII) criteria (NCEP definition) (**B**).

There were, in total, 7824 participants with a mean age of 51.5 years and mean BMI of 24.2, and 65.1% were female (Table 1). The prevalence of diabetes, hypertension, hyperlipidemia, and NAFLD was 3.0%, 34.5%, 35.8%, and 22.7%, respectively. The distributions of the body size phenotypes according to the new definition and NCEP definition are shown in Figure 1. Applying the new definition, the MUHNW phenotype (8.8%) was the least common, while the MHNW (28.7%) and MUHO (21.8%) phenotypes were the most common. The prevalence of each body phenotype differed between the two definitions, especially for those with MHO (14.2% vs. 2.6% using the new definition and the NCEP definition, respectively). The participants with obesity were likely male, had higher WHR, SBP, and DBP and poorer metabolic profiles with higher FPG, HbA1c, ALT, AST, and cholesterol levels (Table 1). Moreover, those with obesity were more likely to be current smokers and more likely to be physically inactive compared with normal-weight and OW participants. Participants with MHO at baseline, compared with MUHO, were younger, were more likely to be female, had better metabolic profile with lower BP, FBG, HbA1c, HOMA-IR, ALT, AST and lipid profile, had higher portion of patients with hypertension, NAFLD and carotid atherosclerosis and were less likely to be a current smoker (Table S1). Compared with MUHNW, people with MHNW were younger, were more likely to be female, had better metabolic profile, had lower prevalence of hypertension and carotid atherosclerosis, were less likely to be a current smoker and more likely to be a current drinker. However, the prevalence of NAFLD was not different between people with MUHNW and MHNW. As for overweight participants, those with MHOW were likely to exercise regularly (≥3/week) than those with MUHOW.

**Figure 2:**
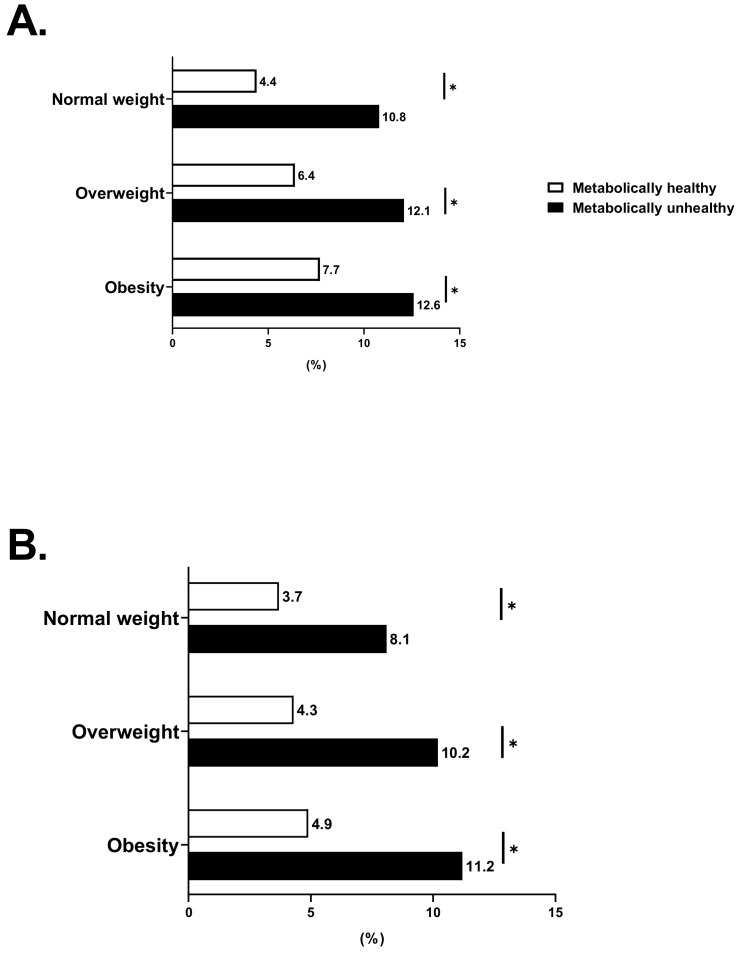
Prevalence of carotid atherosclerosis across categories of weight and metabolic status: based on new definition (**A**) and based on the National Cholesterol Education Program–Adult Treatment Panel III (NCEP-ATPIII) criteria (NCEP definition). (**B**). * *p* < 0.05 compared between metabolically healthy group and metabolically unhealthy group according to body mass index (BMI) category.

**Figure 3:**
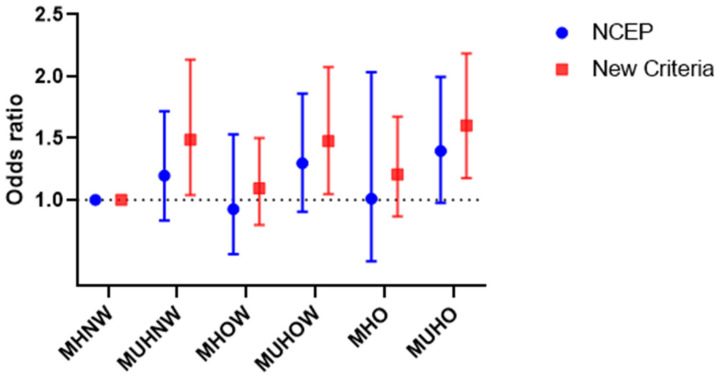
Risk of carotid atherosclerosis in subgroups of body mass index and metabolic health using the new definition (red square) and the National Cholesterol Education Program–Adult Treatment Panel III (NCEP-ATPIII) criteria (NCEP definition) (blue dot). Hazard ratios (95% CIs) adjusted for age, sex, HbA1c, TG, HDL-C, LDL-C, hsCRP, eGFR, HOMA-IR, smoking status, alcohol consumption, physical activity, and the corresponding other definition of metabolic health. Abbreviations: CI, confidence interval; HbA1c, hemoglobin A1c; TG, triglyceride; HDL-C, high-density lipoprotein cholesterol; LDL-C, low-density lipoprotein cholesterol; hs-CRP, high-sensitivity C-reactive protein; eGFR, estimated glomerular filtration rate; HOMA-IR, homeostatic model assessment of insulin resistance; MHNW, metabolically healthy normal weight; MUHNW, metabolically unhealthy normal weight; MHOW, metabolically healthy overweight; MUHOW, metabolically unhealthy overweight; MHO, metabolically healthy obese; MUHO, metabolically unhealthy obese.

When MH was defined by the new criteria, metabolically unhealthy individuals had a higher prevalence of carotid atherosclerosis than metabolically healthy subjects, regardless of BMI category. The prevalence of carotid atherosclerosis (Figure 2) steadily increased across BMI categories among metabolically healthy subjects, whereas for metabolically unhealthy subjects, the extent of the disease was similarly high for OW and OB individuals when compared with normal-weight individuals. After adjustment for age, sex, TG, HDL-C, LDL-C, hsCRP, HbA1c, eGFR, HOMA-IR, smoking status, alcohol consumption, physical activity, and the NCEP definition, all individuals classified as metabolically unhealthy were at increased odds for carotid atherosclerosis independent of BMI categories. Conversely, the odds for the presence of carotid atherosclerosis in individuals with MHO were not significantly increased compared to the MHNW participants (hazard ratio [HR] 1.20, 95% confidence interval [CI] 0.87–1.67) (Figure 3). These associations remained stable despite the adjustment for the NCEP definition (Supplementary Table S1). Furthermore, we subdivided participants with obesity into obesity class 1 (BMI, 25.0–29.9) and 2 (BMI ≥ 30) and repeated the main analysis (Supplementary Figure S1). MHO was not associated with an increased risk of carotid atherosclerosis compared with MHNW for obesity classes 1 and 2 (obesity class 1: HR, 1.22; 95% CI, 0.88–1.70; obesity class 2: HR, 1.12; 95% CI, 0.33–3.75). In subjects with MUHO, the risk of carotid atherosclerosis increased linearly with increasing obesity class (obesity class 1: HR, 1.55; 95% CI, 1.13–2.12; obesity class 2: HR, 2.17; 95% CI, 1.36–3.48; *p* for trend = 0.003) even after adjustment for age, sex, TG, HDL-C, LDL-C, hsCRP, HbA1c, eGFR, HOMA-IR, smoking status, alcohol consumption, physical activity, and the NCEP definition. When MH was defined by the NCEP-ATP III criteria, similar associations were observed only in OW and OB subjects. Moreover, none of the groups displayed increased risks when adjusted for the new definition (Figure 3 and Supplementary Table S1).

**Figure 4:**
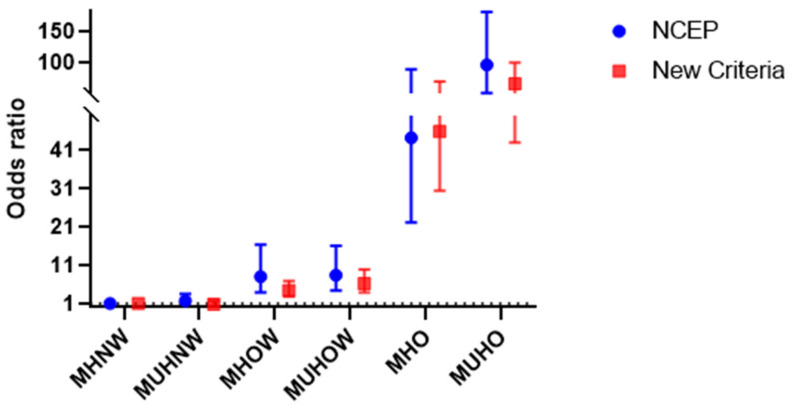
Risk of nonalcoholic fatty liver disease in subgroups of body mass index and metabolic health using the new definition (red square) and the National Cholesterol Education Program–Adult Treatment Panel III (NCEP-ATPIII) criteria (NCEP definition) (blue dot). Hazard ratios (95% CIs) adjusted for age, sex, HbA1c, TG, HDL-C, LDL-C, hsCRP, HOMA-IR, smoking status, AST, ALT, γGTP, smoking status, alcohol consumption, physical activity, and the corresponding other definition of metabolic health. Abbreviations: CI, confidence interval; hemoglobin A1c; TG, triglyceride; HDL-C, high-density lipoprotein cholesterol; LDL-C, low-density lipoprotein cholesterol; hs-CRP, high-sensitivity C-reactive protein; HOMA-IR, homeostatic model assessment for insulin resistance; ALT, alanine transferase; AST, aspartate aminotransferase; GGT, gamma glutamyltransferase; MHNW, metabolically healthy normal weight; MUHNW, metabolically unhealthy normal weight; MHOW, metabolically healthy overweight; MUHOW, metabolically unhealthy overweight; MHO, metabolically healthy obese; MUHO, metabolically unhealthy obese.

When the odds of having NAFLD were investigated, similar associations were observed using both definitions of MH (Figure 4). Individuals classified as OW or OB had increased odds of developing NAFLD, irrespective of MH. In contrast, subjects with normal weight were not at increased odds of developing NAFLD, regardless of MH status. Overall, there was a substantial increase in the risk of NAFLD across increasing BMI categories, and there was an additive effect of being metabolically unhealthy in OW and OB participants.

## 4. Discussion

In this cross-sectional study of a community-based cohort of middle-aged adults without a history of CVD, we applied a new definition of MH to categorize participants as metabolically healthy or unhealthy. Almost 40% of individuals with obesity were classified as MHO within this cohort, and this group was not associated with an increased odds of subclinical atherosclerosis compared with MHNW individuals. Conversely, individuals classified as metabolically unhealthy were at an increased risk compared with MHNW participants, independent of BMI category. The results remained robust even after adjusting for potential confounders including age, sex, TG, HDL-C, LDL-C, hsCRP, HbA1c, HOMA-IR, eGFR, smoking status, alcohol consumption, physical activity, and the NCEP definition. With regard to NAFLD, all individuals classified as OW or OB had an increased risk of NAFLD compared to normal-weight subjects, regardless of MH status.

Previous prospective studies, meta-analyses, and systemic reviews on MHO and CVD have shown conflicting results due to the lack of a uniform definition of the condition, and differences in potential confounders adjusted in analyses [9,10,13,29,30,31,32]. Similarly, in a recent prospective study of UK Biobank participants, people with MHO were at a substantially higher risk of CVD. In the study, they defined the MH with six metabolic markers including BP, C-reactive protein (CRP), triacylglycerols, LDL-cholesterol, HDL-cholesterol and HbA1c [33], while the new definition of MH was derived from the systemic assessment of CVD mortality data and various anthropometric and metabolic factors among individuals with obesity [16]. Many studies have defined MH as the absence of MetSyn, insulin sensitivity, absence of hypertension, diabetes, hyperlipidemia, or any of these metabolic factors. However, this new definition places an emphasis on the WHR and does not account for dyslipidemia. Although waist circumference is commonly used to capture abdominal obesity, a central component of the metabolic syndrome, WHR is a more effective measurement of central adiposity, with WHR having the strongest gradient with incident CVD [34]. In the present study, we applied a new definition of and demonstrated that metabolically unhealthy middle-aged adults without clinical CVD were more likely to have carotid atherosclerosis compared to those who were metabolically healthy. The risk was most significantly elevated in the metabolically unhealthy class 2 obesity group. An increased risk was also observed in metabolically unhealthy individuals within the normal weight category. However, there was no increased risk of subclinical atherosclerosis in the metabolically healthy groups of individuals irrespective of BMI category. Differences in subclinical atherosclerosis were due to MH status, suggesting a major role for MH in subclinical atherosclerosis in comparison with obesity status. Furthermore, in our study, the results remained robust even after adjusting for multiple clinical confounders including hsCRP, HOMA-IR, smoking, and physical activity, which are all well-known risk factors for CVD. However, when MH was defined by the absence of all metabolic parameters of the NCEP-ATP III criteria, this approach did not clearly detect lean but metabolically unhealthy subjects who were at risk for subclinical atherosclerosis. Furthermore, no association was found between body phenotypes and subclinical atherosclerosis after adjusting for the new definition.

The underlying mechanism involved in the MHO phenotype is not fully understood; however, accumulating evidence suggests that less visceral adiposity and less insulin resistance are involved in the MHO phenotype [35]. Adiponectin transgenic (AdTG) leptin-deficient ob/ob mice remained insulin sensitive despite significant weight gain and had a high circulating concentration of adiponectin compared to its ob/ob littermate [36]. This AdTG ob/ob mouse was also found to have increased subcutaneous adipose tissue and low fat content in the liver and skeletal muscle [36]. In another ob/ob mouse model overexpressing the mitochondrial membrane protein mitoNEET, the mice remained insulin sensitive despite significant weight gain [37]. They were also found to have a predominant expansion of subcutaneous fat mass with increased amounts of adiponectin [37]. In our cohort, we had a subset of participants (*n* = 1571) who had adiponectin levels measured at baseline. Consistent with these animal studies, MH individuals who are OW or with obesity had higher levels of adiponectin than metabolically unhealthy individuals who are OW or with obesity (6478.5 vs. 4941.9 and 5592.9 vs. 4712.1 ng/mL, in metabolically healthy vs. unhealthy OW and metabolically healthy vs. unhealthy obesity respectively; *p* < 0.001, data not shown). Although it is beyond the scope of this study, it is plausible that metabolically healthy groups of individuals may have a favorable body fat distribution with higher circulating levels of adiponectin, thus protecting them from CVD. Interestingly, this protection was limited to subclinical atherosclerosis but not fatty liver, which is consistent with a previous study [38].

There was a marked difference in the prevalence of NAFLD between the groups. The prevalence of NAFLD increased substantially with increasing BMI, regardless of MH status. The prevalence of NAFLD in NW, OW and obese participants were 1.3%, 9.0% and 55.0% respectively. These findings are further supported by previous large-scale cohort studies, which found that obesity was strongly associated with incident NAFLD and worsening of liver fibrosis in NAFLD independent of MH [39,40,41]. Although the mechanisms whereby obesity contributes to NAFLD remain unclear, recent studies identified key molecular mediators such as 11β-hydroxysteroid-dehydrogenase 1, fibroblast growth factor-21 and neurotensin, which could potentially contribute to the development of NAFLD in patients with obesity [42,43,44,45]. Currently, emerging epidemiological evidence indicates that MHO may be significantly associated with a greater risk of developing NAFLD [46]. As demonstrated in this study, MHO patients had a higher risk of NAFLD than MHNW individuals, but this risk was generally lower than that observed in MUHO patients, suggesting a greater adverse impact of coexisting metabolic abnormities than obesity alone on the development of NAFLD. Although it is still unclear why the MHO phenotype was not protected from NAFLD but from CVD, study suggests that overweight and obesity are much stronger risk factors for NAFLD than for CVD. Additional studies are also needed to better characterize individuals with MHO who do not develop NAFLD.

We found that the CVD risk among those with the MHO phenotype did not increase compared to that of the MHNW. To date, recommendations for obesity treatment do not consider differences between healthy and unhealthy OW or OB phenotypes. However, the stratification of OB individuals based on their cardiometabolic phenotype may be important for identifying those who are to be prioritized for early pharmacological treatment in addition to lifestyle intervention. Future longitudinal studies are needed to evaluate the impact of lifestyle and treatment interventions on changes in body phenotypes and their effect on the delay or acceleration of subclinical atherosclerosis.

However, this study has several limitations. First, as in all cross-sectional studies, we cannot conclude that there is a causal effect of body phenotype and subclinical atherosclerosis. Although we tried to adjust for known risk factors using multivariate modeling, there may have been residual confounding by unmeasured and measured variables. However, similar results were observed when the same definition of MH was applied to an independent prospective cohort [16]. Secondly, even though this new definition of MH was again able to identify those with MHO without subclinical atherosclerosis, the outcome of the interests differed from the original study by which the definition was derived (CVD mortality and subclinical atherosclerosis). However, several previous studies have demonstrated that carotid atherosclerosis is a marker of systemic atherosclerosis, a powerful predictor of CVD development and even CVD mortality [47,48,49]. Thirdly, there may have been inter- and intra-observer variability in measurement of CIMT as it was performed by multiple observers. To minimize inter-observer variability, a predetermined protocol was used in all clinical centers and inter-rater reliability of CIMT measurements was assessed on sample participants which was excellent [50]. Lastly, our cohort consisted of volunteers not representative of the Korean population and may have limited generalizability. However, our homogenous participants without a previous history of CVD might have strengthened the association between body type and carotid atherosclerosis observed in this study.

This study had several strengths. This study included a large, community-based cohort study of middle-aged Asian participants with a broad spectrum of clinical variables as clinical risk factors. To our knowledge, this is the first study to validate the utility of this new, revised definition of MH by evaluating associations with carotid atherosclerosis, in comparison with other definitions in an Asian population without clinical CVD.

## 5. Conclusions

In conclusion, with a new and simple definition of MH, we were able to classify people with MHO who are not at an increased risk for subclinical atherosclerosis. This new definition was also helpful in detecting lean people at risk. Routine screening of CVD risk among the metabolically unhealthy, regardless of BMI category, may provide an opportunity to institute intensive pharmacological therapy to prevent subsequent CVD.

## Figures and Tables

**Table 1:** Baseline Characteristics of the Study cohort.

	CMERC Cohort (*n* = 7824)	Normal Weight (*n* = 2934)	Overweight (*n* = 2068)	Obesity (*n* = 2822)	*p*
Age (years)	51.52 ± 8.63	50.64 ± 8.86	52.40 ± 8.30	51.78 ± 8.55	**<0.001**
Sex (Female)	5095 (65.1%)	2287 (77.9%)	1336 (64.6%)	1472 (52.2%)	**<0.001**
BMI (kg/m^2^)	24.2 ± 3.0	21.4 ± 1.1	24.0 ± 0.6	27.4 ± 2.2	**<0.001**
WHR	0.67 ± 0.09	0.61 ± 0.06	0.66 ± 0.07	0.73 ± 0.08	**<0.001**
SBP (mmHg)	119.9 ± 15.4	114.5 ± 14.8	120.5 ± 14.3	125.1 ± 14.9	**<0.001**
DBP (mmHg)	76.8 ± 10.2	73.4 ± 9.5	76.9 ± 9.5	80.2 ± 10.1	**<0.001**
FPG (mg/dl)	95.7 ± 20.7	90.9 ± 16.7	95.4 ± 20.2	100.9 ± 23.4	**<0.001**
HbA1c (%)	5.7 ± 0.7	5.5 ± 0.6	5.7 ± 0.7	5.9 ± 0.8	**<0.001**
HOMA-IR	1.9 [ 1.5; 2.6]	1.60 [1.28; 2.00]	1.92 [1.50; 2.50]	2.45 [1.85; 3.36]	**<0.001**
ALT (U/L)	25.2 ± 18.5	20.6 ± 13.1	23.9 ± 13.3	31.0 ± 24.2	**<0.001**
AST (U/L)	25.7 ± 13.5	24.3 ± 9.8	25.0 ± 7.6	27.7 ± 18.9	**<0.001**
γGTP (IU/L)	31.3 ± 43.2	23.2 ± 26.8	29.4 ± 29.7	41.1 ± 60.1	**<0.001**
eGFR (mL/min/1.73 m^2^)	85.1 ± 13.5	86.4 ± 13.2	84.5 ± 13.6	84.3 ± 13.6	**<0.001**
Total cholesterol (mg/dL)	195.2 ± 34.7	193.3 ± 33.4	195.2 ± 34.7	197.3 ± 36.0	**<0.001**
Triglyceride (mg/dL)	132.5 ± 93.6	106.0 ± 61.5	133.0 ± 91.4	159.6 ± 113.0	**<0.001**
HDL-C (mg/dL)	55.7 ± 14.0	60.7 ± 14.6	54.6 ± 13.3	51.3 ± 12.1	**<0.001**
LDL-C (mg/dL)	115.3 ± 31.2	112.4 ± 29.7	116.1 ± 30.5	117.8 ± 33.0	**<0.001**
Hs-CRP (mg/L)	0.58 [0.33; 1.19]	0.42 [0.27; 0.77]	0.57 [0.34; 1.10]	0.82 [0.45; 1.66]	**<0.001**
Current smoking (%)	2416 (30.9%)	629 (21.4%)	634 (30.7%)	1153 (40.9%)	**<0.001**
Current drinking (%)	5839 (74.6%)	2186 (74.5%)	1542 (74.6%)	2111 (74.8%)	0.964
Physical activity (days/week)					**<0.001**
None	4627 (59.1%)	1656 (56.4%)	1264 (61.1%)	1707 (60.5%)	
<3	1375 (17.6%)	498 (17.0%)	336 (16.2%)	541 (19.2%)	
≥3	1822 (23.3%)	780 (26.6%)	468 (22.6%)	574 (20.3%)	
Diabetes (%)	237 (3.0%)	7 (0.2%)	20 (1.0%)	210 (7.4%)	**<0.001**
Hypertension (%)	2703 (34.5%)	601 (20.5%)	737 (35.6%)	1365 (48.4%)	**<0.001**
Hyperlipidemia (%)	1253 (35.8%)	346 (31.0%)	342 (36.0%)	565 (39.3%)	**<0.001**
NAFLD (%)	1778 (22.7%)	40 (1.4%)	187 (9.0%)	1551 (55.0%)	**<0.001**
Mean IMT (mm)	0.62 [0.55; 0.70]	0.59 [0.54; 0.67]	0.62 [0.56; 0.70]	0.64 [0.57; 0.73]	**<0.001**
Carotid atherosclerosis (%)	653 (8.3%)	172 (5.9%)	179 (8.7%)	302 (10.7%)	**<0.001**
Metabolic phenotype					**<0.001**
Metabolically healthy	4613 (59.0%)	2249 (76.7%)	1251 (60.5%)	1113 (39.4%)	
Metabolically unhealthy	3211 (41.0%)	685 (23.3%)	817 (39.5%)	1709 (60.6%)	

Data are presented as mean ± standard deviation, median (interquartile range), or number (%). Values with statistical significance are shown in bold. Abbreviations: BMI, body mass index; WHR, hip ratio; SBP, systolic blood pressure; DBP, diastolic blood pressure; FPG, fasting plasma glucose; HbA1c, hemoglobin A1c; HOMA-IR, homeostatic model assessment for insulin resistance; ALT, alanine transferase; AST, aspartate aminotransferase; GGT, gamma glutamyltransferase; eGFR, estimated glomerular filtration rate; HDL-C, high-density lipoprotein cholesterol; LDL-C, low-density lipoprotein cholesterol; Hs-CRP, high-sensitivity C-reactive protein; NAFLD, nonalcoholic fatty liver disease; IMT, intima-media thickness.

## Data Availability

The data presented in this study are available on request from the corresponding author.

## Supplementary Materials

The following supporting information can be downloaded at: https://www.mdpi.com/article/10.3390/jcm11092440/s1, Figure S1: Risk of subclinical atherosclerosis in subgroups classified by BMI categories extended by obesity class 1 to 2 and metabolic health defined by a new definition; Table S1: Odds ratios for carotid atherosclerosis in subgroups of body mass index and metabolic health using the new definition and the NCEP definition. Table S2: Odds ratios for carotid atherosclerosis in subgroups classified by BMI categories extended by Obesity Class 1 to 2 and metabolic health using the new definition and the NCEP definition.
