# Supplementary material for: Association between Metabolically Healthy Obesity and Subclinical Atherosclerosis in the Cardiovascular and Metabolic Diseases Etiology Research Center (CMERC) Cohort
Source: J Clin Med. 2022 Apr 26;11(9):2440. doi: 10.3390/jcm11092440 (PMC9103721; doi:10.3390/jcm11092440)
Supplement: Supplementary file 1 [file jcm-11-02440-s001.zip › jcm-1662518-supplementary.pdf]

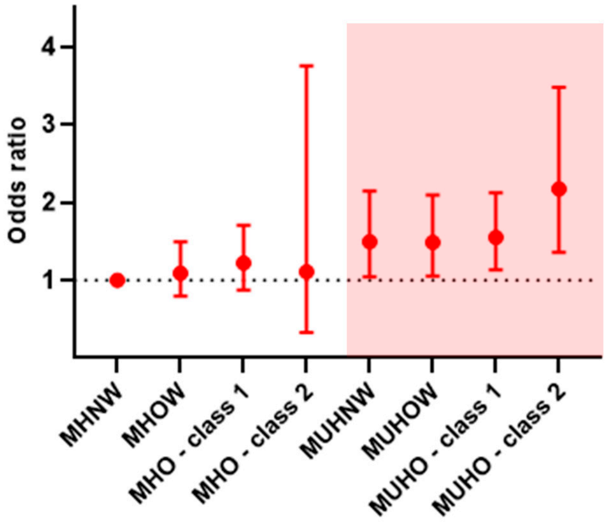

Figure S1. Risk of subclinical atherosclerosis in subgroups classified by BMI categories extended by obesity class 1 to 2 and metabolic health defined by a new definition. Hazard ratios (95% CIs) adjusted for age, sex, HbA1c, TG, HDL-C, LDL-C, hsCRP, eGFR, HOMA-IR, smoking status, alcohol consumption, physical activity, and other definition of metabolic health by the National Cholesterol Education Program–Adult Treatment Panel III (NCEP-ATPIII) criteria. BMI, body mass index; CI, confidence interval; HbA1c, hemoglobin A1c; TG, triglyceride; HDL-C, high-density lipoprotein cholesterol; LDL-C, low-density lipoprotein cholesterol; hs-CRP, high-sensitivity C-reactive protein; eGFR, estimated glomerular filtration rate; HOMA-IR, homeostatic model assessment of insulin resistance; MHNW, metabolically healthy normal weight; MUHNW, metabolically unhealthy normal weight; MHOW, metabolically healthy overweight; MUHOW, metabolically unhealthy overweight; MHO, metabolically healthy obese; MUHO, metabolically unhealthy obese.

Table S1. Baseline Characteristics of the Study cohort according to obesity and metabolic health status.

	Normal weight (N=2934)			Overweight (N=2068)			Obese (N=2822)		
	**MH (N=2249)**	**MUH (N=685)**	**p**	**MH (N=1251)**	**MUH (N=817)**	**p**	**MH (N=1113)**	**MUH (N=1709)**	**p**
Age (years)	49.2 ± 9.0	55.3 ± 6.4	< **0.001**	50.9 ± 8.6	54.8 ± 7.2	< **0.001**	50.4 ± 8.7	52.7 ± 8.3	< **0.001**
Sex (Female)	1828 (81.3%)	459 (67.0%)	< **0.001**	871 (69.6%)	465 (56.9%)	< **0.001**	670 (60.2%)	802 (46.9%)	< **0.001**
BMI (kg/m^2)	21.29 ± 1.15	21.62 ± 1.06	< **0.001**	23.94 ± 0.57	24.06 ± 0.56	< 0.001	26.73 ± 1.54	27.77 ± 2.41	< **0.001**
WHR	0.60 ± 0.06	0.62 ± 0.06	< **0.001**	0.66 ± 0.06	0.67 ± 0.07	< 0.001	0.70 ± 0.07	0.75 ± 0.08	< **0.001**
SBP (mmHg)	109.3 ± 9.5	131.6 ± 16.1	< **0.001**	113.6 ± 8.9	131.2 ± 14.3	< **0.001**	115.8 ± 8.3	131.2 ± 15.1	< **0.001**
DBP (mmHg)	70.8 ± 7.6	81.9 ± 10.3	< **0.001**	73.2 ± 7.2	82.6 ± 9.8	< **0.001**	75.2 ± 7.0	83.4 ± 10.5	< **0.001**
FPG (mg/dl)	87.9 ± 9.1	100.5 ± 28.3	< **0.001**	90.8 ± 10.2	102.4 ± 28.3	< **0.001**	93.4 ± 12.0	105.8 ± 27.3	< **0.001**
HbA1c (%)	5.4 ± 0.3	5.9 ± 1.0	< **0.001**	5.5 ± 0.4	5.9 ± 0.9	< **0.001**	5.6 ± 0.4	6.0 ± 1.0	< **0.001**
HOMA-IR	1.6 [1.3; 1.9]	1.7 [1.4; 2.3]	< **0.001**	1.8 [1.4; 2.3]	2.1 [1.6; 2.8]	< **0.001**	2.2 [1.7; 2.9]	2.7 [2.0; 3.7]	< **0.001**
ALT (U/L)	19.8 ± 12.5	23.1 ± 14.9	< **0.001**	22.9 ± 13.2	25.4 ± 13.2	< **0.001**	27.4 ± 16.9	33.4 ± 27.8	< **0.001**
AST (U/L)	23.8 ± 7.8	26.2 ± 14.4	< **0.001**	24.7 ± 7.8	25.4 ± 7.3	**0.029**	26.0 ± 10.4	28.9 ± 22.8	< **0.001**
γGTP (IU/L)	21.4 ± 22.6	29.1 ± 36.9	< **0.001**	27.1 ± 29.1	32.9 ± 30.2	< **0.001**	32.8 ± 30.8	46.4 ± 72.6	< **0.001**
eGFR (mL/min/1.73 m^2)	87.2 ± 13.4	83.8 ± 12.4	< **0.001**	85.5 ± 13.3	82.9 ± 14.0	< **0.001**	84.8 ± 13.6	83.9 ± 13.6	0.079
Total cholesterol (mg/dL)	193.4 ± 33.1	192.9 ± 34.5	0.732	197.5 ± 34.7	191.6 ± 34.4	< **0.001**	201.0 ± 33.4	194.8 ± 37.3	< **0.001**
Triglyceride (mg/dL)	100.9 ± 57.1	122.8 ± 71.8	< **0.001**	125.9 ± 92.4	143.9 ± 88.8	< **0.001**	142.6 ± 88.1	170.7 ± 125.4	< **0.001**
HDL-C (mg/dL)	61.3 ± 14.5	58.5 ± 14.6	< **0.001**	55.6 ± 13.3	53.1 ± 13.2	< **0.001**	52.9 ± 12.0	50.2 ± 12.0	< **0.001**
LDL-C (mg/dL)	112.7 ± 29.2	111.3 ± 31.3	0.321	118.6 ± 30.3	112.4 ± 30.4	< **0.001**	121.9 ± 31.1	115.2 ± 34.0	< **0.001**
Hs-CRP (mg/L)	0.4 [0.3; 0.8]	0.5 [0.3; 0.9]	**0.001**	0.5 [0.3; 1.0]	0.6 [0.4; 1.2]	< **0.001**	0.7 [0.4; 1.4]	0.9 [0.5; 1.9]	< **0.001**
Current smoking (%)	427 (19.0%)	202 (29.5%)	< **0.001**	345 (27.6%)	289 (35.4%)	< **0.001**	374 (33.6%)	779 (45.6%)	< **0.001**
Current drinking (%)	1697 (75.5%)	489 (71.4%)	**0.037**	939 (75.1%)	603 (73.8%)	0.556	826 (74.2%)	1285 (75.2%)	0.590
Physical activity (days/week)									
None	1263 (56.2%)	393 (57.4%)	0.102	757 (60.5%)	507 (62.1%)	**0.009**	660 (59.3%)	1047 (61.3%)	
<3	369 (16.4%)	129 (18.8%)		186 (14.9%)	150 (18.4%)		207 (18.6%)	334 (19.5%)	0.169
≥3	617 (27.4%)	163 (23.8%)		308 (24.6%)	160 (19.6%)		246 (22.1%)	328 (19.2%)	
Diabetes (%)	0 (0.0%)	7 (1.0%)	< **0.001**	0 (0.0%)	20 (2.4%)	< **0.001**	0 (0.0%)	210 (12.3%)	< **0.001**
Hypertension (%)	0 (0.0%)	601 (87.7%)	< **0.001**	0 (0.0%)	737 (90.2%)	< **0.001**	0 (0.0%)	1365 (79.9%)	< **0.001**
Hyperlipidemia (%)	195 (29.7%)	151 (33.0%)	0.270	153 (38.0%)	189 (34.6%)	0.310	142 (40.6%)	423 (38.9%)	0.625
NAFLD (%)	29 (1.3%)	11 (1.6%)	0.662	87 (7.0%)	100 (12.2%)	< **0.001**	509 (45.7%)	1042 (61.0%)	< **0.001**
Mean IMT (mm)	0.58 [0.53;0.65]	0.65 [0.57;0.73]	< **0.001**	0.61 [0.55;0.68]	0.66 [0.58;0.74]	< **0.001**	0.61 [0.56;0.70]	0.66 [0.59;0.74]	< **0.001**
Carotid atherosclerosis (%)	98 (4.4%)	74 (10.8%)	< **0.001**	80 (6.4%)	99 (12.1%)	< **0.001**	86 (7.7%)	216 (12.6%)	< **0.001**

Data are presented as mean ± standard deviation, median (interquartile range), or number (%).Values with statistical significance are shown

in bold. Abbreviations: MH, metabolically healthy; MUH, metabolically unhealthy; BMI, body mass index; WHR, hip ratio; SBP, systolic blood pressure; DBP, diastolic blood pressure; FPG, fasting plasma glucose; HbA1c, hemoglobin A1c; HOMA-IR, homeostatic model assessment for insulin resistance; ALT, alanine transferase; AST, aspartate aminotransferase; GGT, gamma glutamyltransferase; eGFR, estimated glomerular filtration rate; HDL-C, high-density lipoprotein cholesterol; LDL-C, low-density lipoprotein cholesterol; Hs-CRP, high-sensitivity C-reactive protein; NAFLD, nonalcoholic fatty liver disease; IMT, intima-media thickness.

Table S2. Odds ratios for carotid atherosclerosis in subgroups of body mass index and metabolic health using the new definition and the NCEP definition.

	**Normal weight**		**Overweight**		**Obese**	
	**MH**	**MUH**	**MH**	**MUH**	**MH**	**MUH**
**New definition**						
**Model 1**	1	**1.76 (1.28–2.43)**	1.28 (0.94–1.75)	**1.92 (1.42–2.59)**	1.53 (1.13–2.07)	**2.14 (1.66–2.77)**
**Model 2**	1	**1.68 (1.21–2.33)**	1.13 (0.83–1.55)	**1.65 (1.21–2.25)**	1.30 (0.95–1.76)	**1.78 (1.35–2.36)**
**Model 3***	1	**1.49 (1.04–2.13)**	1.09 (0.80–1.50)	**1.47 (1.05–2.089)**	1.20 (0.87–1.67)	**1.60 (1.18–2.19)**
**NCEP**						
**Model 1**	1	**1.66 (1.18–2.32)**	1.00 (0.61–1.66)	**1.96 (1.42–2.71)**	1.12 (0.56–2.26)	**2.20 (1.63–2.98)**
**Model 2**	1	1.41 (1.00–1.99)	0.93 (0.56–1.53)	**1.54 (1.09–2.17)**	1.01 (0.50–2.04)	**1.70 (1.22–2.37)**
**Model 3 †**	1	1.20 (0.83–1.72)	0.93 (0.56–1.53)	1.30 (0.90–1.86)	1.01 (0.50–2.03)	1.40 (0.98–2.00)

All models were constructed using logistic regression analysis. Model 1: adjustment for age and sex; model 2: model 1 + adjustment for HbA1c, TG, HDL-C, LDL-C, hsCRP, eGFR, HOMA-IR, smoking status, alcohol consumption, and physical activity; model 3: model 2 + adjustments for NCEP definition ***** or new definition of metabolic health. NCEP, National Cholesterol Education Program–Adult Treatment Panel III; HbA1c, hemoglobin A1c; TG, triglyceride; HDL-C, high-density lipoprotein cholesterol; LDL-C, low-density lipoprotein cholesterol; hs-CRP, high-sensitivity C-reactive protein; eGFR, estimated glomerular filtration rate; HOMA-IR**,** homeostatic model assessment for insulin resistance; MH, metabolically healthy; MUH, metabolically unhealthy.
